# Virtual reality environments for post-stroke arm rehabilitation

**DOI:** 10.1186/1743-0003-4-20

**Published:** 2007-06-22

**Authors:** Sandeep Subramanian, Luiz A Knaut, Christian Beaudoin, Bradford J McFadyen, Anatol G Feldman, Mindy F Levin

**Affiliations:** 1 School of Physical and Occupational Therapy, McGill University, 3654 Promenade Sir William Osler, Montreal, H3G 1Y5, Canada; 2 School of Rehabilitation, University of Montreal, C.P. 6128, Succursale Centre-Ville Montreal, H3C 3J7, Canada; 3 CRIR Research Center, Jewish Rehabilitation Hospital, 3205 Alton Goldbloom Place, Laval, H7V 1R2, Canada; 4 Department of Rehabilitation, Laval University, Ste Foy, G1K 7P4, Canada; 5 Department of Physiology, University of Montreal, C.P. 6128, Succursale Centre-Ville Montreal, H3C 3J7, Canada

## Abstract

**Introduction:**

Optimal practice and feedback elements are essential requirements for maximal motor recovery in patients with motor deficits due to central nervous system lesions.

**Methods:**

A virtual environment (VE) was created that incorporates practice and feedback elements necessary for maximal motor recovery. It permits varied and challenging practice in a motivating environment that provides salient feedback.

**Results:**

The VE gives the user knowledge of results feedback about motor behavior and knowledge of performance feedback about the quality of pointing movements made in a virtual elevator. Movement distances are related to length of body segments.

**Conclusion:**

We describe an immersive and interactive experimental protocol developed in a virtual reality environment using the CAREN system. The VE can be used as a training environment for the upper limb in patients with motor impairments.

## Background

Stroke, third leading cause of death in Western countries, contributes significantly to disabilities and handicaps. Up to 85% of patients have an initial arm sensorimotor dysfunction with impairments persisting for more than 3 months [1,2]. Several principals guide motor recovery. In animal stroke models, experience-dependent plasticity is driven through salient, repetitive and intensive practice [3,4]. However, in humans, unguided practice of reaching without feedback about movement patterns used, even if enhanced or intensive, may reinforce compensatory movement strategies instead of encouraging recovery of pre-morbid movement patterns [5,6]. While desirable for some patients with severe impairment and poor prognosis, for others, compensation may limit the potential for recovery [7-10].

Levin and colleagues have shown that recovery of pre-morbid movement patterns after repetitive reaching training is facilitated when either compensatory trunk movements were restricted [11] or information about missing motor elements was provided [6,12]. This suggests that more salient, task-relevant feedback may result in greater motor gains after stroke. Virtual reality (VR) technologies provide adaptable media to create environments for assessment and training of arm motor deficits using enhanced feedback [13]. This paper describes a virtual environment (VE) that incorporates practice and feedback elements necessary for maximal motor recovery. It introduces: 1) *originality and motivation *to the task; 2) *varied and challenging practice *of high-level motor control elements, and 3) *optimal, multimodal feedback *about movement performance and outcome.

## Methods

**Figure 1:**
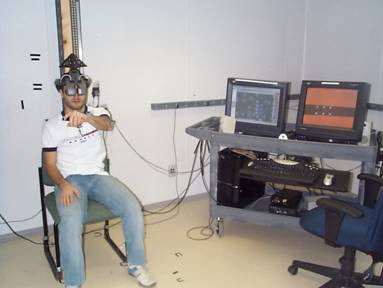
A subject performing the experiment (left) beside the virtual reality system (right).

A VE simulating elevator buttons was developed to practice pointing movement (Fig. 1). Target placement challenges individuals to reach into different workspace areas and motivation is provided as feedback about motor performance. Peripherals are connected to a PC (Dual Xeon 3.06 GHz, 2 GB RAM, 160 GB hard drive) running a CAREN (Computer Assisted Rehabilitation Environment; Motek BV) platform providing 'real-time' integration of 3D hand, arm and body position data with the VE. The system includes a head-mounted display (HMD, Kaiser XL50, resolution 1024 × 768, frequency 60 Hz), an Optotrak Motion Capture System (Northern Digital), a CyberGlove^® ^(Immersion), and a dual-head Nvidia Quatro FX3000 graphics card (70 Hz) providing high-speed stereoscopic representation of the environment created on SoftImage XSI.

The 3D visual scene displayed through the HMD promotes a sense of presence in the VE [14]. To simulate stereovision, two images of the same environment are generated in each HMD camera position with an offset corresponding to inter-ocular distance. The Optotrak system tracks movement in the virtual space via infrared emitting diodes (IREDs) placed on body segments. Optotrak provides higher sampling rates and shorter latencies for acquiring positional data compared to other systems, e.g., electromagnetic. Longer latencies may be associated with cybersickness. Head and hand position are determined by tracking rigid bodies on the HMD and CyberGlove respectively.

Presence is enhanced with the 22-sensor CyberGlove, permitting the user to see a realistic reproduction of his/her hand in the VE. Haptic feedback is not provided (i.e., force feedback on button depression). Hand position from Optotrak tracking is relayed to CyberGlove software, which calculates palm and finger position/orientation. Final fingertip position determines target acquisition with accuracy adjusted to the participant's ability.

### Experimental Setup

**Figure 2:**
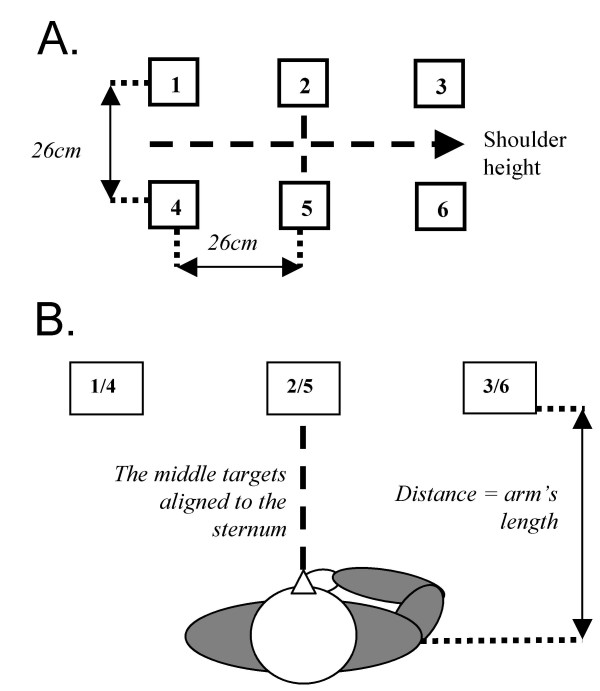
Target arrangement on coronal (A) and transversal planes (B).

The system permits repetitive training of goal-directed arm movements to improve arm motor function. In the current setup, elevator buttons (targets), displayed in 2 rows of 3, 6 cm × 6 cm targets (Fig. 2), are arranged on a virtual wall in the ipsilateral and contralateral arm workspace requiring different combinations of arm joint movements for successful pointing. Center-to-center distance between adjacent targets is 26 cm (Fig. 2A). Targets are displayed at a standardized distance equal to the participant's arm length (Fig. 2B) to facilitate collision detection. Middle targets are aligned with the sternum, with the mid-point between rows at shoulder height.

**Figure 3:**
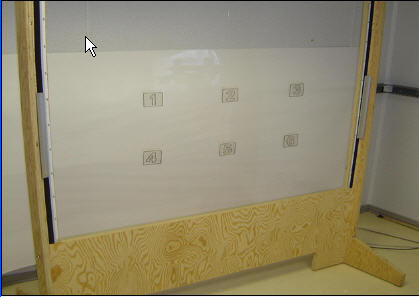
Physical target grid for virtual environment calibration.

**Figure 4:**
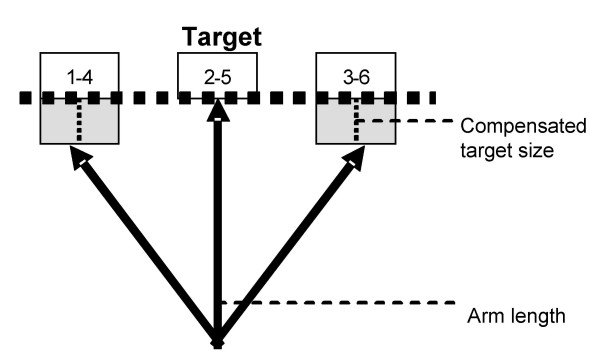
Compensation of target size along the sagittal direction taking into account the arc of the arm.

A global system axis is calibrated using a grid of physical targets having the exact size and relative position as those in the VE, with its origin at the center of the target grid (Fig. 3). Extreme right and left target distances (1,4,3,6) are corrected for arm's length by offsetting target depth along the sagittal plane (Fig. 4) so that they can be reached without trunk displacement.

Based on findings that improvement in movement time of a reaching task occurred after 25–35 trials in patients with mild-to-moderate hemiparesis [7], the initial training protocol includes 72 trials. This represents twice the number needed for motor learning and is considered intensive. Trials are equally and randomly distributed across targets. Twelve trials per target are recorded, 3 blocks of 24 movements each, separated by rest periods. Recording time and intertrial intervals are adjusted according to subject ability. Task difficulty is progressed by manipulating movement speed and precision requirements.

### Feedback

**Figure 5:**
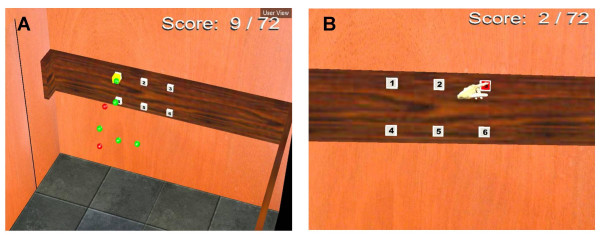
Elevator scenes: A. Spheres represent marker positions on the subject's arm and trunk and the cube in front of Target 1 is the offset added to detect collision between the fingertip and the target. B. The virtual environment as it appears to the subject in the head-mounted display. The subject is cued to reach Target 3. The participant's score is indicated on the top right of each panel.

Effects of different types of feedback on motor learning can be studied. Feedback is provided as knowledge of results (KR) and performance (KP). Movement speed and precision (KR) and motor performance (joint movement patterns, KP) auditory and visual feedback is provided to enhance motor learning [6,12]. Subjects are verbally cued to reach to a target as well as by a change in target color (yellow, Fig. 5A,B). Subjects receive positive feedback (KR) in the form of a 'ping' sound and change in target color (green) when the movement is both within the stipulated time and area. Negative feedback (buzzer sound) is provided if the movement is not rapid or precise enough. Finally, the subject receives KP in the form of a 'whoosh' sound and red colored target if trunk displacement exceeds an adjustable default value of 5 cm. According to previous studies, non-disabled subjects use up to 1.7 ± 1.6 cm of trunk movement to reach similarly placed targets [15].

## Preliminary Results

**Figure 6:**
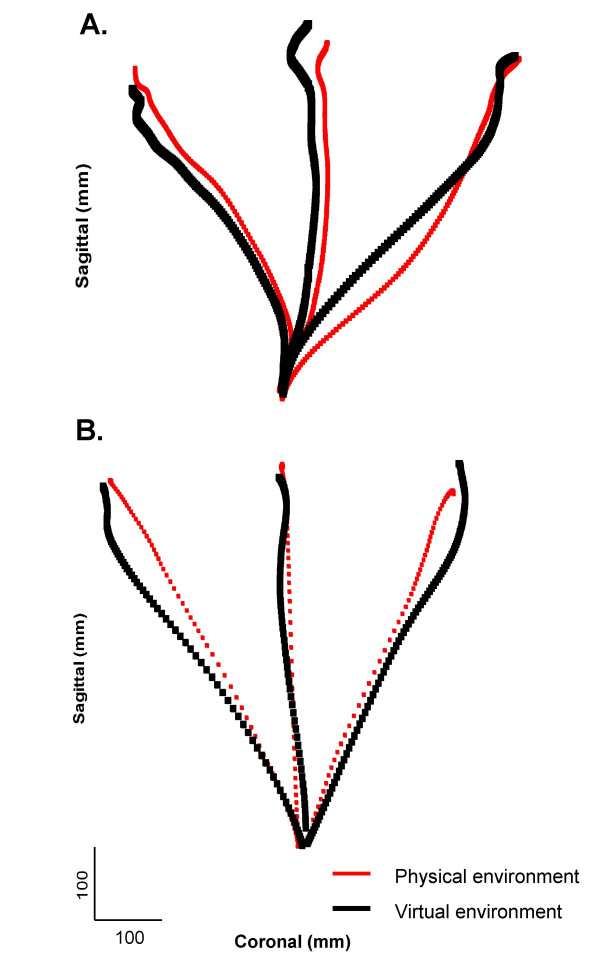
Endpoint trajectories of the pointing movement performed in the physical environment (thin lines, red) and the virtual environment (thick lines, black) by a patient with hemiparesis (A) and a non-disabled subject (B).

We compared motor performance and movement patterns made to the 6 targets between the VE and PE (Fig. 6) in 15 patients with hemiparesis and 8 age-matched non-disabled controls. Position data (x, y, z) from the finger, arm and trunk were interpolated and filtered and trajectories were calculated. Kinematics measured were endpoint velocity, pointing error and trajectory smoothness. Peak endpoint velocity was determined from magnitude of the tangential velocity obtained by differentiation of index marker positional data. Endpoint error was calculated as the root-mean-square error of endpoint position with respect to the target. Trajectory smoothness was computed as the curvature index defined as ratio of actual endpoint path length to a straight line joining starting and end positions such that a straight line has an index of 1 and a semicircle has an index of 1.57 [16].

Fig. 6 shows mean endpoint trajectories for one patient with moderate hemiparesis (A) and one non-disabled subject (B) reaching to the 3 lower targets in both environments. The non-disabled subject made movements twice as fast as the patient. In both subjects, movement speed was lower in the VE. Endpoint precision was comparable, ranging from 257–356 mm in the PE and 275–370 mm in the VE for the non-disabled subject and from 263–363 mm in the PE and 275–379 mm in the VE for the patient. Movements tended to be less precise and more curved in VE compared to the PE (curvature index: non-disabled-PE: 1.02–1.03; VE: 1.04–1.05; patient-PE: 1.15–1.22; VE: 1.16–1.32). Results suggest some differences in movements performance in a VE compared to a PE of similar physical dimensions. From a usability standpoint, only 2 patients of those screened could not use the HMD. Of those who participated, all reported that the VE was more enjoyable and motivating than the PE and it encouraged them to do more practice.

## Conclusion

A VR system was developed to study effects of enhanced feedback on motor learning and arm recovery in patients with neurological dysfunction. Effects will be contrasted with those from practice in similarly constructed PEs using different types of feedback.

## References

[B1] Carod-Artal J, Egido JA, Gonzalez JL, Varela de Seijas E (2000). Quality of life among stroke survivors evaluated 1 year after stroke: experience of a stroke unit. Stroke.

[B2] Olsen TS (1990). Arm and leg paresis as outcome predictors in stroke rehabilitation. Stroke.

[B3] Teasell R, Bayona NA, Bitensky J (2005). Plasticity and reorganization of the brain post stroke. Top Stroke Rehabil.

[B4] Nudo RJ, Milliken G (1996). Reorganization of movement representations in primary motor cortex following focal ischemic infarcts in adult squirrel monkeys. J Neurophysiol.

[B5] Cirstea MC, Levin MF (2000). Compensatory strategies for reaching in stroke. Brain.

[B6] Cirstea MC, Ptito A, Levin MF (2006). Effect of type of feedback and cognitive impairment in arm motor skill re-acquisition in stroke. Stroke.

[B7] Allred RP, Maldonado MA, Hsu JE, Jones TA (2005). Training the "less-affected" forelimb after unilateral cortical infarcts interferes with functional recovery of the impaired forelimb in rats. Restor Neurol Neurosci.

[B8] Taub E, Miller NE, Novack TA (1993). Technique to improve chronic motor deficit after stroke. Arch Phys Med Rehab.

[B9] Ada L, Canning C, Carr JH, Kilbreath SL, Shepherd RB, Bennett KMB, Castiello U (1994). Task specific training of reaching and manipulation. Insights into Grasp and Reach Movements.

[B10] Levin MF (1997). Should stereotypic movement synergies seen in hemiparetic patients be considered adaptive?. Behav Brain Sci.

[B11] Michaelsen SM, Dannenbaum R, Levin MF (2006). Task-specific training with trunk restraint on arm recovery in stroke: randomized control trial. Stroke.

[B12] Cirstea MC, Levin MF (2007). Improvement in arm movement patterns and endpoint control depends on type of feedback during practice in stroke survivors. Neurorehabil Neural Repair.

[B13] Stanton D, Foreman N, Wilson PN (1998). Uses of virtual reality in clinical training: developing the spatial skills of children with mobility impairments. Stud Health Technol Informatics.

[B14] McNeill MDJ, Pokluda L, McDonough SM, Crosbie J (2004). Immersive virtual reality for upper limb rehabilitation following stroke. Proceedings of IEEE International Conference on Systems, Man and Cybernetics.

[B15] Levin MF, Cirstea MC, Michaelsen SM, Roby-Brami A (2002). Use of trunk for reaching targets placed within and beyond the reach in adult hemiparesis. Exp Brain Res.

[B16] Archambault P, Pigeon P, Feldman AG, Levin MF (1999). Recruitment and sequencing of different degrees of freedom during pointing movements involving the trunk in healthy and hemiparetic subjects. Exp Brain Res.

